# Seasonal Patterns in the Prevalence and Diversity of Tick-Borne *Borrelia burgdorferi* Sensu Lato, *Anaplasma* *phagocytophilum* and *Rickettsia* spp. in an Urban Temperate Forest in South Western Slovakia

**DOI:** 10.3390/ijerph15050994

**Published:** 2018-05-15

**Authors:** Michal Chvostáč, Eva Špitalská, Radovan Václav, Tatiana Vaculová, Lenka Minichová, Markéta Derdáková

**Affiliations:** 1 Institute of Zoology, Slovak Academy of Sciences, Dúbravská cesta 9, 845 06 Bratislava, Slovakia; michal.chvostac@gmail.com (M.C.); radovan.vaclav@savba.sk (R.V.); tana.vaculova@gmail.com (T.V.); 2 Institute of Virology, Biomedical Research Center, Slovak Academy of Sciences, Dúbravská cesta 9, 845 05 Bratislava, Slovakia; eva.spitalska@savba.sk (E.Š.); lenka.berthova@savba.sk (L.M.)

**Keywords:** *Borrelia burgdorferi*, *Anaplasma phagocytophilum*, *Rickettsia*, *Ixodes ricinus*, host, temporal variation

## Abstract

In Europe, *Ixodes ricinus* is the most important vector of tick-borne zoonotic bacteria. It transmits spirochaetes from the *Borrelia burgdorferi* sensu lato complex, *Anaplasma phagocytophilum* and *Rickettsia* spp. Although spatial differences in the prevalence of tick-borne pathogens have been intensively studied, seasonal (within-year) fluctuations in the prevalence of these pathogens within sites are often overlooked. We analyzed the occurrence and seasonal dynamics of *Ixodes ricinus* in an urban forest in Bratislava, Slovakia. Furthemore, we examined temporal trends in the community structure of *B. burgdorferi* s.l., *A. phagocytophilum* and *Rickettsia* spp. in questing and bird-feeding ticks. The total prevalence for *B. burgdorferi* s.l. in questing *I. ricinus* was 6.8%, involving six genospecies with the dominance of bird-associated *B. garinii* and *B. valaisiana.* *A. phagocytophilum*, *R. helvetica* and *R. monacensis* occurred in 5.9%, 5.0% and 0.2% of questing ticks, respectively. In total, 12.5% and 4.4% of bird-feeding *I. ricinus* ticks carried *B. burgdorferi* s.l. and *R. helvetica*. The total prevalence of *B. burgdorferi* s.l. in our study site was two times lower than the mean prevalence for Europe. In contrast, *A. phagocytophilum* prevalence was significantly higher compared to those in other habitats of Slovakia. Our results imply that tick propagation and the transmission, suppression and seasonal dynamics of tick-borne pathogens at the study site were primarily shaped by abundance and temporal population fluctuations in ruminant and bird hosts.

## 1. Introduction

*Ixodes ricinus* (Linnaeus 1758) is the most widespread tick species in Europe. It widely occurs in sylvatic as well as in urban habitats with ample vegetation such as parks, recreational areas, gardens and urban forests. Emergence of ticks in urban areas is associated with a higher risk of tick-borne diseases for humans and companion animals [1]. Occurrence of ticks in urban parks is significantly influenced by vegetation that maintains adequate humidity for ticks as well as the abundance and species composition of hosts [2,3,4].

*I. ricinus* ticks transmit several bacterial agents. The most common ones are spirochetes from the *Borrelia burgdorferi* sensu lato complex. This bacterial complex consists of 21 known genospecies [5,6,7,8,9,10,11,12]. Five out of eight genospecies that are present in Europe are potentially infectious for humans and can cause Lyme disease. 

Reported mean prevalence of *B. burgdorferi* s.l., detected by various detection methods including polymerase chain reaction (PCR), cultivation in BSK medium, dark-field microscopy and immunofluorescence assays, in ticks in Europe is 12.3–13.7% [13,14]. In Slovakia, the prevalence ranges from 4.5%, detected in questing *I. ricinus* in a suburban forest in northern Slovakia [15], to 46.1% for a mountain region of central Slovakia [16]. Prevalences of this bacterial group in urban and suburban forests across various cities in Slovakia were recorded to reach 20.5% [15,17,18]. Genetic variability within and between *Borrelia* genospecies is linked to different clinical symptoms as well as different associations to reservoir hosts. Rodents, especially from genera *Myodes* and *Apodemus* act as the reservoirs of *B. afzelii*, *B. burgdorferi* s.s., *B. bavariensis*, and *B. spielmanii* [19,20,21,22,23]. The circulation of *B. afzelii* in natural foci of urban parks and forests is also associated with squirrels and hedgehogs [20,24], while *B. spielmanii* is associated with hedgehogs [24] and dormice [25]. Birds are the reservoirs of *B. garinii*, *B. valaisiana* [26] and in a low degree also for *B. lusitaniae* [27] for which the main reservoir hosts are the lizards [28]. 

*Anaplasma phagocytophilum* is another important bacterial agent transmitted by *I. ricinus* in Europe. It causes granulocytic anaplasmosis of humans, horses and dogs, as well as tick-borne fever of domestic ruminants. In Slovakia, the prevalence of *A. phagocytophilum* in questing *I. ricinus* ticks varies between 1.1% and 8% [15,29,30,31,32]. In Europe, *Anaplasma* circulates in two different cycles. The most important reservoir hosts in Europe are wild cervids, sheep, cattle and goats [29,33,34,35,36]. Rodents are reservoir hosts of *A. phagocytophilum* genotypes that are transmitted by *I. trianguliceps*, but not by *I. ricinus* ticks [36,37,38,39]. In areas with abundant populations of wild cervids, the prevalence of *Anaplasma* is usually higher [34,39].

*Ixodes ricinus* ticks are also known to carry some species of *Rickettsia*, obligate, aerobic, gram-negative, intracellular bacterial parasites of eukaryotes with a worldwide occurrence, which can cause rickettsial diseases. *Rickettsia helvetica* and *Rickettsia monacensis* transmitted by *I. ricinus* are rickettsial species associated with human cases of rickettsiosis [40,41]. The prevalences vary from 0.5% in an island in the Baltic Sea to 66% in the Netherlands for *R. helvetica*, and from 0.5% in Germany to 34.6% in Turkey for *R. monacensis* [42]. In Slovakia, rickettsial prevalence in *I. ricinus* ticks were recorded up to 20%, with *R. helvetica* being a dominant species [43,44]. The role of vertebrates in their circulation is still not clear. Rickettsiae have been identified in a few randomly selected samples of the spleens of small rodents and ungulates [45,46]. 

All of *I. ricinus* stages are obligate parasites. Immature stages of ticks feed most frequently on small mammals and birds. Howeverd, the most important host of adult ticks are large mammals, such as deer and wild boar. Immature ticks can compensate for the absence of birds and small mammals by feeding on larger animals, but their abundance may decrease [47,48].

The population structure of tick hosts in different sites and habitats can affect the abundance of tick-borne pathogens such as *Borrelia* and *Anaplasma* due to differences in host competence [49,50,51]. For example, roe deer commonly acts as the main host for adult ticks and in a lower degree for immature ticks [52]. The increase of roe deer abundance may cause a decrease in *Borrelia* prevalence in a given habitat [39], possibly due to the borreliacidal effects of deer blood-complement [53]. Variation in the abundance and diversity of tick hosts has been suggested as a crucial determinant of the prevalence and density of tick-borne pathogens [54]. For example, some might suggest that densities of infected ticks should be lower at sites with higher tick-host biodiversity [55], but see [56]. However, the abundance and diversity of tick hosts and, consequently, the abundance and diversity of tick-borne pathogens, may not only vary spatially, but also temporally within sites, e.g., due to fluctuating densities of bird tick hosts. Despite its importance for epidemiology, seasonality and its causes in the prevalence and community structure of vector-borne pathogens within sites have been scarcely studied (e.g., [57] for prevalence of *B. burgdorferi* s.l. and *A. phagocytophilum* during May and August). 

The aim of this study was to examine the seasonal dynamics in *I. ricinus* abundance as well as the prevalence and community structure of *B. burgdorferi* s.l., *A. phagocytophilum* and *Rickettsia* spp. in an urban forest in Slovakia. A recent metaanalysis has revealed a strong negative correlation between the two most prevalent *Borrelia* genospecies in Europe—*B. afzelii* and *B. garinii* [14], suggesting that small rodents and birds represent the main alternative hosts for immature *I. ricinus* ticks in the region. Given the abundance of temperate-zone birds undergoes abrupt seasonal changes corresponding to their reproductive period (e.g., [58]), we hypothesized that the seasonal pattern in the prevalences of tick-borne pathogens at the study site would reflect the phenology of bird abundance. Consequently, we predicted that the prevalence of the commonest *Borrelia* genospecies associated with bird hosts (*B. garinii*) and small rodent hosts (*B. afzelii*) should show divergent seasonal patterns, reflecting the breeding phenology of temperate-zone birds.

## 2. Materials and Methods

### 2.1. Study Area, Tick Sampling and Host Trapping

The study area (app. 0.15 km^2^) was located in NW Bratislava, Slovakia (48°10′11.303″ N; 17°4′2.255″ E), in the Sitina urban forest. The forest is a part of the Slovak Academy of Sciences (SAS) campus. It is largely separated from the Bratislava ZOO and from the surrounding urban area by the fence. The study site is characterized by a low abundance of small rodents [59]. On the other hand, many temperate-zone bird species, mainly passerines, which harbour the majority of *Borrelia*-infected ticks [60], such as tits (Paridae) and thrushes (Turdidae) are seasonally abundant there during their reproductive period (April–July). The local breeding populations of these passerine species are migratory (wintering in Western Europe and the Mediterranean region [61]) and show higher numbers during the breeding period compared to the winter season. Some large vertebrates, particularly roe deer *C. capreolus*, are also permanently present at the site. In addition, a population of app. 35 feral cats resides in the study area. Hornbeams *Carpinus betulus* and oaks *Quercus* spp. dominate the forest.

Questing ticks were collected by standardized flagging method [62] in monthly intervals from March 2011 until December 2012 on a single 100 m transect. Various types of vegetation were covered during flaging. Birds were caught during four trappings throughout the summer season in 2012 by mist-nets [60], and feeding ticks were removed from birds with forceps. To confirm the presence or absence of small mammals, five two-night trappings with 50 Swedish bridge metal traps [63] were conducted during 2011–2012. All the questing and feeding ticks were stored in 70% ethanol until the DNA extraction. Only nymphal and adult stages were used for the investigation. Ticks were identified to species and life stages using available taxonomic keys [64]. Meteorological data for the days of tick collection were acquired from the station of the Hydrometeorological Institute (Bratislava, Mlynská dolina) that is located 2 km away from the study site.

### 2.2. DNA Extraction and PCR Detection of Bacteria

A total of 543 questing ticks and 295 bird-fed *I. ricinus* ticks were tested for the prevalence of tick-borne bacteria. DNA was isolated from ticks individually. For questing ticks, alcaline-hydrolysis method [65] was used. DNA from bird-fed ticks was isolated with the commercial extraction kit (DNAeasy tissue kit, Qiagen, Hilden, Germany). DNA samples were stored at −20 °C. A 620 bp fragment of tick mitochondrial gene cytochrome b was amplified in each extracted tick DNA to confirm the presence of tick DNA [66]. Only positive samples were further analyzed for the presence of tick borne agents.

### 2.3. Detection of Borrelia burgdorferi s.l.

*B. burgdorferi* s.l. was detected by the amplification of 222–255 bp fragment of rrfA-rrlB intergenic spacer using IGSA (5′-CGACCTTCTTCGCCTTAAAGC-3′) and IGSB (5′-AGCTCTTATTCGCTGATGGTA-3′) primers [67]. PCR (polymerase chain reaction) amplifications were performed in a total reaction mixture of 25 µL. The PCR reaction mixture per each sample contained 2.5 µL of PCR buffer, 1 µL of MgCl_2_, 0.125 µL of polymerase (Qiagen, Hilden, Germany), 0.5 µL of both primers, 0.5 µL of dNTP (Thermofisher, Dreieich, Germany), 14.875 µL of nuclease-free water (Promega, Madison, WI, USA) and 5 µL of tick DNA as template. Touch-down PCR program consisted of these steps: Initial denaturation at 95 °C for 5 min, followed by 5 cycles of denaturation at 94 °C for 15 s, annealing at 61 °C (−0.2 °C per cycle) for 25 s and elongation at 72 °C for 30 s. It was followed by 5 cycles of denaturation at 94 °C for 15 s, annealing at 60 °C (−0.4 °C per cycle) for 25 s and elongation at 72 °C for 30 s. Then followed 30 cycles of denaturation (94 °C, 15 s), annealing (58 °C, 25 s) and elongation (72 °C, 30 s). The program was terminated by elongation at 72 °C for 5 min. The PCR products were electrophoresed on 1.5% agarose gel. Than it was stained with GoodView (Ecoli, Bratislava, Slovakia) and visualized on UV transiluminator Vilber-Lourmant (Sigma-Aldrich, St. Louis, MO, USA). Positive samples were further typed to Borrelial genospecies by Restriction Fragment Length Polymorphism (RFLP) [67]. For each of 13 µL of positive PCR product 0.5 µL of TruI restriction enzyme (Fermentas, Thermo Scientific, Vilnius, Lithuainia) and 1.5 µL of buffer (Fermentas, Thermo Scientific, Vilnius, Lithuania) were mixed and digested for 5 min at 65 °C. The electrophoresis was performed in electrophoresis system Origins (Elchrom Scientific, Cham, Switzerland) using Spreadex EL300 mini gel (Elchrom Scientific, Cham, Switzerland) at 120 V for 150 min. After the electrophoresis, the gel was stained by SYBR green (Sigma-Aldrich, St. Louis, MO, USA) for 45 min and visualized by UV transiluminator. 

### 2.4. Detection of A. phagocytophilum

Samples were tested for the presence of *A. phagocytophilum* by real-time PCR using primers ApMSP2f (5′-ATGGAAGGTAGTGTTGGTTATGGTATT-3′) and ApMSP2r (5′-TTGGTCTTGAAGCGCTCGTA-3′) and the TaqmanProbe ApMSP2p (5′-TGGTGCCAGGGTTGAGCTTGAGATTG-3′, labeled with FAM targeted 77 bp fragment of msp2 gene [68]. As master mix, 12.5 µL of Bioron SuperHot Master Mix was mixed with 0.3 µL of probe, 2.6 µL of MgCl_2_ (Bioron, Ludwigshafen, Germany) and 2.3 µL of both primers. 5 µL of DNA was added into each sample. The PCR program consisted of initial denaturation at 95 °C for 2 min, followed by 39 cycles of denaturation at 95 °C for 15 s and annelation at 60 °C for 1 min. Real-time PCR was calibrated using serial dilution of standard reference sample. Based on the results, a standard curve was defined. Samples were considered positive with threshold cycles (CT) level less than 35 cycles and the amplified products at the same dissociation temperature as the positive control. CT value observed in positive samples ranged from 30 to 35 cycles. 

### 2.5. Detection of R. helvetica and R. monacensis

Samples were tested for the presence of rickettsiae using the genus-specific primers RpCS.877p (5′-GGGGACCTGCTCACGGCGG-3′) and RpCS.1258n (5′-ATTGCAAAAAGTACAGTGAACA-3′) amplifying a 381 bp part of *gltA* gene [69]. PCR amplifications were carried out using DyNAzyme^TM^ PCR MAster Mix (Finnzymes, Espoo, Finland) on thermocycler PTC-119 200 Peltier Thermal Cycler (MJ Research, Saint Bruno, Canada). Master mix composed of following reagents: 10 µL of 2× DyNAzyme^TM^ II PCR Master Mix, 2 µL of both primers, and 5 µL of nuclease-free water (Promega, Madison, WI, USA). 3 µL of DNA was used for PCR. The DNA from uninfected ticks and sterile water, and DNA from *R. helvetica* were used as negative and positive controls, respectively. PCR products were analyzed by electrophoresis in a 1% agarose gel, stained with GelRedTM (Biotium, Hayward, CA, USA) and visualized with the UV transilluminator. Rickettsia-positive tick samples were screened for the presence of *R. helvetica* using TagMan PCR assay targeting a 65-bp fragment of the 23S rRNA gene [70] using DyNAmo^TM^ Probe qPCR (Finnzymes, Espoo, Finnland) on Bio-Rad CFX96^TM^ Real-Time System. As master mix, 10 µL of 2× Master Mix was mixed with 0.25 µL of TaqMan probe (5′-AACCGTAGCGTACACTTA-3′ labeled with TAMRA) and 0.5 µL of both primers Rickhelv.147f (5′-TTTGAAGGAGACACGGAACACA-3′) and Rickhelv.211r (5′-TCCGGTACTCAAATCCTCACGTA-3′). 3 µL of DNA was added into each sample. The PCR program consisted of initial denaturation at 95 °C for 15 min, followed by 40 cycles of denaturation at 95 °C for 15 s and annelation at 60 °C for 1 min. Each run of TaqMan PCR reactions included a negative template control, a positive control, and DNA standards containing 3 × 10^0^–3 × 10^6^ target copies with a sensitivity of 3 copies of the DNA. *R. helvetica*-negative samples by TagMan PCR assay were purified using a QIAquick Spin PCR Purification Kit (Qiagen, Hilden, Germany) as described by the manufacturer and analyzed by sequencing both DNA strands by Marcogen Inc., (Amsterdam, The Netherlands). The DNA sequences were compared with those available in GenBank using the Basic Local Alignment Search Tool (Blast) on http://blast.ncbi.nlm.nih.gov/.

### 2.6. Statistical Analysis

Ordination analysis is a suitable approach to examine multivariate data [71]. In order to examine how seasonality (within-year temporal variation) contributes to explaining the variation in the occurrence of tick-borne pathogens, we used the partial redundancy analysis (partial RDA). The month when *I. ricinus* ticks were collected represented the explanatory variable (constraint) while the life-cycle stage (nymph or adult), the sex of adult ticks, and the year of sampling represented the conditioning (random) variable. In partial RDA, each *I. ricinus* tick represented a unique site (*n* = 542) at which the presence or absence of selected pathogens was studied. The presence-absence data of pathogen occurrence were Hellinger-transformed before the partial RDA analysis [71]. *R. monacensis*, *B. burgdorferi* s.s., and *B. spielmanii* were excluded from RDA analysis because they occurred rarely (in less than four ticks). The significance of partial RDA parameters (RDA axes and the constraint, i.e., month) was tested by permutation tests (999 permutations). The vegan package v. 2.4-1 [72] was used for partial RDA analysis within the R software [73]. Contingency tables with the prevalence data of different pathogens were examined with the likelihood-ratio (G) test, using the RVAideMemoire package v. 0.9-62 [74].

### 2.7. Nucleotide Sequence Accession Numbers

The GenBank accession numbers for the nucleotide sequences of *R. helvetica* and *R. monacensis* obtained in this study are: MF673859-MF673863.

## 3. Results

In total, 559 questing ticks were collected in 2011 and 2012. Sixteen ticks were determined as *Haemaphysalis concinna* Koch, 1844 and these were not included in further analyses. 543 ticks were identified as *Ixodes ricinus*; 249 and 294 *I. ricinus* ticks were collected in 2011 and 2012, respectively. Of these, 484 (89.1%) individuals were nymphs and 59 (10.9%) were adults. Adult ticks represented 2.4% of collected ticks in 2011, while it was 17.7% in 2012. 

During 5 trappings (500 trap-nights), we did not catch any rodent. Out of total 34 mist-netted birds, 309 feeding *I. ricinus* ticks (20 nymphs and 289 larvae) were collected; 295 (20 nymphs and 275 larvae) of them were tested for the presence of bacterial DNA (Table 1 and Table 2). We have readily observed, but not sampled, other vertebrates, particularly roe deer, feral cats and hedgehogs, which can act as hosts of *I. ricinus* ticks in the study area. 

### 3.1. Pathogens in Questing I. ricinus

*Borrelia burgdorferi* s.l. was detected in 37 (6.8%) out of 543 questing *I. ricinus* ticks. Two (3.4%) of 58 *I. ricinus* adults and 35 (7.2%) of 485 nymphs were positive for *B. burgdorferi* s.l. (Table 3).

**Figure 1:**
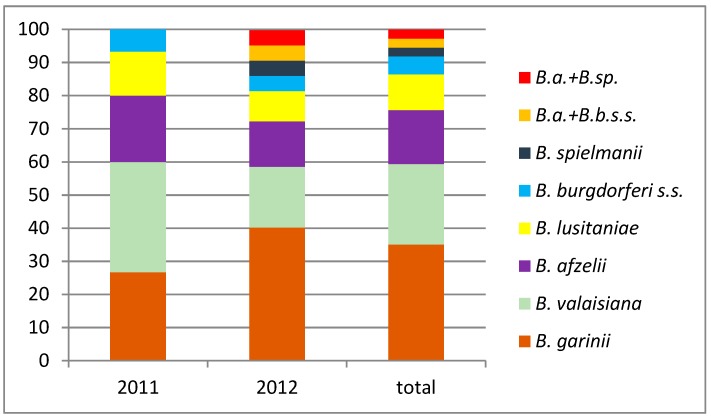
Proportions of different genospecies of *B. burgdorferi* s.l. detected in questing *I. ricinus* ticks for the urban forest in Bratislava, Slovakia, 2011–2012.

In 37 borrelia-positive samples, we have detected the presence of six genospecies with the dominance of *B. garinii* (13 ticks, 35.1%) and *B. valaisiana* (9 ticks, 24.3%). *B. afzelii* was present in six ticks (16.2%). *B. lusitaniae* was a less prevalent genospecies (4 ticks, 10.8%), followed by *B. burgdorferi* s.s. (2 ticks, 5.4%) and *B. spielmanii* (1 tick, 2.7%). Mixed infection was recorded in two cases. One tick was infected with *B. afzelii* and *B. spielmanii* and the other one with *B. afzelii* and *B. burgdorferi* s.s. (Figure 1).

*A. phagocytophilum* was detected in 32 (5.9%) out of 543 questing ticks (Table 3). *A. phagocytopphilum* prevalence in 2011 was 4.4% (11 ticks), while in 2012 it was 7.1% (21 ticks) (Table 3). The infection rate of adult *I. ricinus* was comparable (3 ticks; 5.2%) with the *A. phagocytophilum* prevalence in nymphal *I. ricinus* (29 ticks; 6%). 

*R. helvetica* and *R. monacensis* were identified in 27 (5.0%) and one (0.2%) questing tick, respectively (Table 3). *R. helvetica* was found in 15 specimens (6%; CI: 3.4–9.7) out of ticks collected in 2011 and in 12 (7.5%; CI: 4.7–11.1) out of ticks collected in 2012. *R. monacensis* was recorded in one female collected in June 2012. The prevalence in 2012 was 0.3%, while the total prevalence of *R. monacensis* was 0.18% (CI: 0.0–1.0). All *R. helvetica*-positive adults were recorded in 2012 only, while 8.0% (14/176) and 3.7% (9/242) of nymphs were positive for the pathogen in 2011 and 2012, respectively. Co-infections involving *R. helvetica* were recorded with *A. phagocytophilum*, *B. lusitaniae*, *B. garinii*, and *B. valaisiana* in four nymphs collected in 2011 and with *A. phagocytophilum* and *B. valaisiana* in two nymphs collected in 2012. 

### 3.2. Pathogens in Bird-Feeding I. ricinus

In total, 12.5% (37/295) bird-feeding *I. ricinus* ticks were borrelia-positive (Table 1 and Table 2), with the dominance (97%) of *B. garinii*. A single nymph, collected from *Parus major* carried *B. afzelii*. *Borrelia* prevalence in nymphs and larvae was 15% (3/20) and 12.4% (34/275), respectively.

None of the bird-feeding ticks carried *A. phagocytophilum*. Thirteen (4.4%) out of 295 tested bird-feeding ticks carried *R. helvetica*. One (5%) out of 20 nymphs and 12 (4.4%) out of 275 larvae were positive. *R. monacensis* was not detected in any of the ticks feeding on birds (Table 1 and Table 2).

### 3.3. Seasonality in Tick Abundance and Pathogen Occurrence

**Figure 2:**
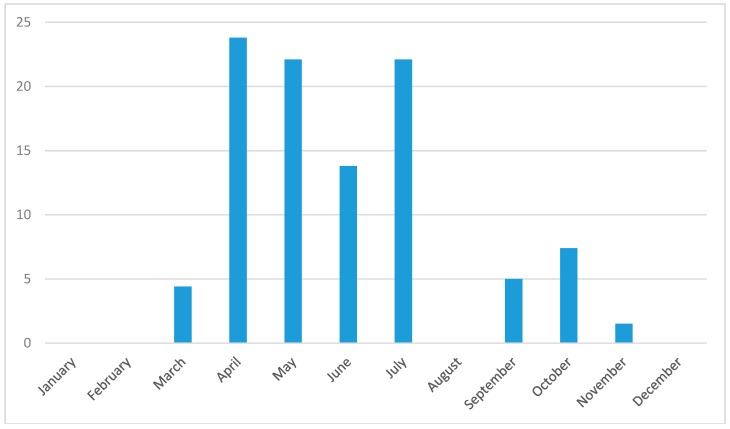
Relative abundance of *I. ricinus* ticks for the urban forest in Bratislava, Slovakia, during individual months over two study years, 2011–2012. Relative abundance means the proportion (%) of ticks collected during a certain month divided by the total number of collected ticks. Note that ticks were not sampled during January and February.

**Figure 3:**
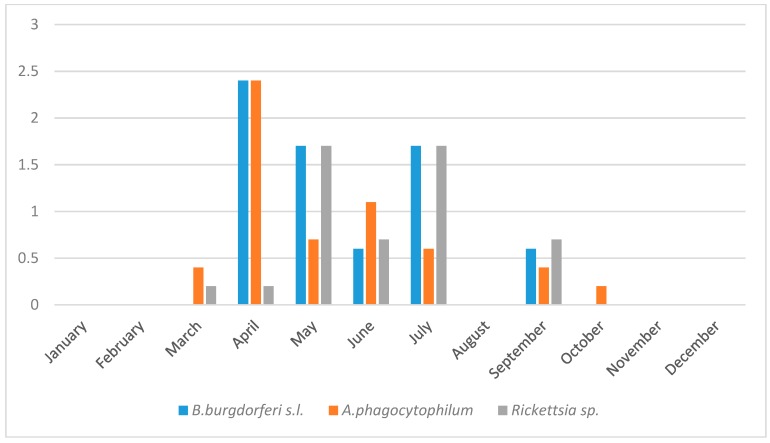
Relative prevalence of *B. burgdorferi* s.l., *A. phagocytophilum*, *Rickettsia* sp. in questing *I. ricinus* ticks for the urban forest in Bratislava, Slovakia, during individual months over two study years, 2011–2012. Relative prevalence means the proportion (%) of positive ticks for the given pathogen during a certain month divided by the total number of collected ticks. Note that ticks were not sampled during January and February.

The seasonal dynamics of tick questing activity showed a bimodal pattern with the first peak in April–May and the second peak in July (Figure 2). The highest abundance of ticks was recorded in April, which corresponed with the highest prevalence of *B. burgdorferi* s.l in questing ticks (Figure 3). Despite a relatively low abundance of questing ticks during September, *B. burgdorferi* s.l. was still present in questing ticks (Figure 3). The prevalence of *A. phagocytophilum* peaked in April (Figure 3). Rickettsiae were most prevalent in May and July (Figure 3).

**Figure 4:**
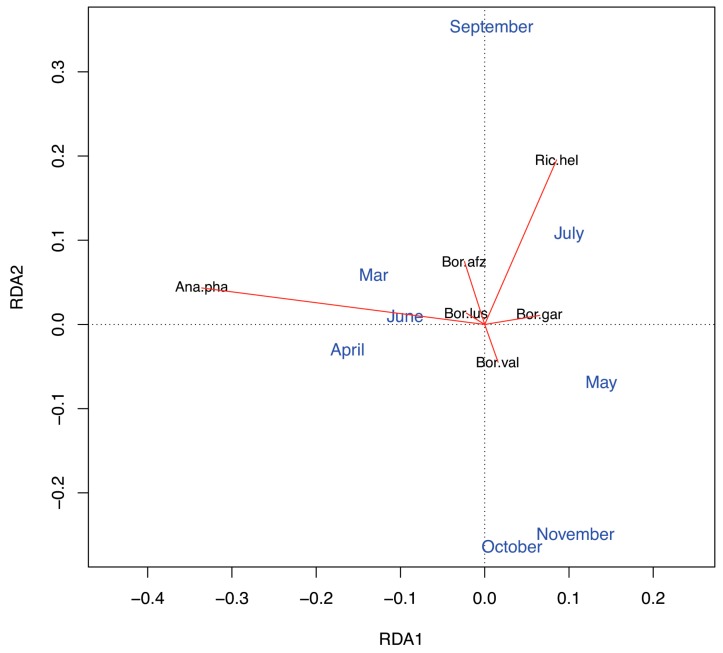
Partial redundancy analysis (partial RDA) examining the occurrence of selected tick-borne pathogens constrained by the effect of seasonality (month of sampling). The partial RDA biplot (scaling 2) shows the scores of pathogens (black letters and red arrows) and the centroids of the months of the year (blue letters). The contribution (unadjusted proportions) of three components of the partial RDA to total inertia explained: Conditioning variables (tick life-cycle stage, sex, and year) = 0.003, constraining variable (month of sampling) = 0.02, and unconstrained variables = 0.97. Pathogens shown in the biplot are *Anaplasma phagocytophilum* (Ana.pha), *Rickettsia helvetica* (Ric.hel), *Borrelia lusitaniae* (Bor.lus), *B. afzelii* (Bor.afz), *B. garinii* (Bor.gar), and *B. valaisiana* (Bor.val).

The partial redundancy analysis (partial RDA) revealed a significant effect of season in terms of month of sampling on the occurrence of selected pathogens in questing *I. ricinus* ticks (month of sampling, F = 1.78, df = 7, *p* < 0.01). *A. phagocytophillum* was more likely to occur in March and April than in other months (Figure 4). While *B. afzelii* more likely occurred in September and less likely in October and November, this was just the opposite for *B. valaisiana* (Figure 4). *B. garinii* occurrence was particularly likely in May and less likely in April (Figure 4). *R. helvetica* typically occurred in July, but also in September. Variation in the occurrence of pathogens in questing ticks was significantly arranged along two distinct gradients (i.e., constrained axes). The partial RDA suggests that the occurrence of *A. phagocytophilum* diverged from that of *B. garinii* (RDA1, F = 7.68, df = 1, *p* < 0.001), and the occurrence of *R. helvetica* and *B. afzelii* was dissociated from that of *B. valaisiana* (RDA2, F = 2.93, df = 1, *p* = 0.018; Figure 4). 

## 4. Discussion

Changes in biotic and abiotic conditions can increase or decrease the transmission of zoonotic diseases [4]. Variation in tick-borne pathogen prevalence is traditionally viewed in the context of spatial variation in climatic and weather conditions [75] or spatial variation, at different scales, in tick-host community structure [54]. In this study we have investigated questing activity of ticks and their infection with three groups of tick-borne pathogens—*Borrelia*, *Anaplasma* and *Rickettsia*. For an urban temperate forest, we examined whether the prevalence of different tick-borne pathogens varies temporally within a season. We hypothesized that the occurrence of the commonest *Borrelia* genospecies in questing ticks should vary non-randomly in time with respect to the reproductive period of birds. This hypothesis is relevant because our study site is known to have a low abundance of small rodents [53], thus stressing the role of bird hosts for local immature *I. ricinus* ticks. We found that the occurrence of different tick-borne pathogens varied non-randomly with time of season. 

Tick questing activity showed a bimodal seasonal pattern. In both years, the first peak was recorded in April–May and the second one in July. This bimodal seasonal tick activity with one bigger peak in spring and the second one in early fall has been described for various tick habitats in Central Europe [76,77,78,79]. The overall prevalence of tick-borne pathogens followed the temporal trend in tick abudance. However, by focusing on specific tick-borne pathogens, we detected distinct seasonal trends in the occurrences of the most common pathogens (Figure 4). First, the early season (March–May) was characterised by the diverging occurrence of *A. phagocytophilum* and *B. garinii*. Second, the late season (September–November) was characterised by the divergent occurrence of *B. afzelii* and *B. valaisiana*. Hence, even though *B. afzelii* and *B. garinii* typically occurred at different parts of the year, our results do not support a divergent occurrence of *B. afzelii* and *B. garinii* at our study site. We suggest that this finding is due to a locally lower importance of small rodent hosts during the part of the year with the highest tick abundance (see below). Nevertheless, this study indicates that small rodents may still be important alternative tick hosts during some periods, because we found a divergent occurrence of *B. afzelii* and *B. valaisiana* during the late season. Importantly, our results lend support to our hypothesis that temporary increases in the abundance of bird hosts during their reproductive period can be reflected by the seasonal trends in the occurrence of the pathogens associated with these hosts, namely, *B. garinii*. Intriguingly, the occurrence of *B. garinii* in questing ticks was most likely in May, i.e., during the peak reproductive period of birds. Even though we do not possess knowledge of the duration of tick molt at our study site, our results suggest that the cohorts of questing *I. ricinus* ticks infected by bird-associated *B. garinii* are likely to transmit this pathogen to the same host group. Further research is necessary to address how seasonal fluctuations in abiotic factors, such as temperature and humidity, contribute to the seasonal trends in tick-borne pathogen occurrence and, specifically, to the synchronisation between the occurrence of infected ticks and the corresponding host reservoirs of infection.

*A. phagocytophilum* was detected in 5.9% of questing ticks. Prevalences of *A. phagocytophilum* in questing *I. ricinus* ticks in Slovakia and neigbouring European countries vary between 0.57% and 13.1% [30,37,59,80,81,82]. As compared to recent works on *A. phagocytophilum* prevalence for various urban and sylvatic habitats in Slovakia [30,37,59], the pathogen prevalence was significantly higher for our urban forest study area (Table 4).

*Anaplasma phagocytophilum* shows a considerable genetic variability [83], which is partly explained by reservoir host specificity. In Europe, *A. phagocytophilum* consists of two genetically distinct ecotypes that circulate in two enzootic cycles—one involving rodents and *I. trianguliceps* ticks and the other involving ungulates, carnivores, and insectivores and *I. ricinus* ticks [37,84,85,86]. Moreover, Blaňárová et al. [37] showed that *I. ricinus* ticks could not contract *A. phagocytophilum* while feeding on infected rodents, though *I. trianguliceps* ticks could contract the pathogen from infected rodents. Consequently, roe deer and hedgehogs do not only appear to represent the main host group for ticks, but also the main reservoir hosts of *A. phagocytophilum* in the study area.

The circulation of the causative agents of Lyme borreliosis, spirochetes of *B. burgdorferi* s.l. complex, depends on the presence of vector ticks and competent reservoir hosts. In our study we detected the presence of *B. burgdorferi* s.l. in 6.8% (37/543) ticks. The prevalence of this pathogen complex for questing ticks in our study was significantly lower than those detected for various habitats in Slovakia including urban as well as natural sites [26,87] (Table 5). 

In addition, the mean prevalence of *B. burgdorferi* s.l. in *I. ricinus* across Europe (12.3–13.7%) [13,14] is about twice as high as that detected in our study area. Finally, our results on the pathogen prevalence are at the lower range limit for prevalences reported for neighbouring countries (4–25.7%) [67,88,89,90,91,92]. 

The low prevalence of *B. burgdorferi* s.l. was perhaps the best reflected by the low prevalence of *B. afzelii*; this rodent-associated genospecies was present only in 16.2% of borrelia-positive questing ticks. For example, at a different site in Slovakia, Derdáková et al. [67] detected the presence of *B. afzelii* in 56.5% of positive ticks. Rauter and Hartung [13] revealed that on average 38% of borrelia-positive ticks across Europe are infected with *B. afzelii*. Rodents represent the most important host group for the maintenance of juvenile *I. ricinus* in Central and Eastern Europe [93] and the key reservoir hosts for *B. afzelii, B. spielmanii, B. bavariensis* and *B. burgdorferi* s.s. [19,21,22,25]. Importantly, the presence of *B. afzelii*, *B. burgdorferi* s.s. or *B. spielmanii* can also be maintained in reservoirs, such as hedgehogs and squirrels, especially in urban areas [20,24]. Consequently, the low infection rate of *B burgdorferi* s.l., and particularly low prevalences of *B. afzelii*, *B. spielmanii*, and *B. burgdorferi* s.s., support the findings of a previous study [53] about a low abundance of small mammals at our study site. In fact, given the pattern of *B. garinii* vs. *A. phagocytophilum* and *B. valaisiana* vs. *B. afzelii* occurrences (RDA1 and RDA2 axes in Figure 4), our results indicate that birds and roe deer represented main tick hosts at our study site. Morevover, given the pattern of RDA1 axis, reptiles appear to have served as alternative tick hosts during the periods of low bird abundance (Figure 4). Importantly, ruminants such as roe deer may not only be an incompetent reservoir host for borrelia, but due to the borreliacidal effect of their blood complement, they can eliminate borrelia in feeding ticks [39,53,94]. Overall, the low prevalence of *B. burgdorferi* s.l. points to locally low densities of rodents, but also to the fact that birds and other small vertebrate hosts were not able to compensate for the lack of small rodents. Thus, roe deer appear to constitute one of the main host groups for *I. ricinus* ticks and an important biotic factor behind the abundance and community structure of tick-borne pathogens at the study area.

*B. garinii* (35.1% prevalence) and *B. valaisiana* (24.3% prevalence), which are associated with bird reservoirs, were found to be the locally dominant genospecies of *B. burgdorferi* s.l. in questing ticks. Of trapped birds, 12.5% of bird-feeding ticks carried *B. burgdorferi* s.l. (Table 1) with the almost exclusive occurrence of *B. garinii*. Tarageľová et al. [26] detected 9.4% to 31.1% prevalence of *B. burgdorferi* s.l. in bird-feeding ticks for different natural habitats in Slovakia. In that study, only 44% of borrelia-positive bird-feeding ticks carried *B. garinii*, which was significantly less than we detected at our study site (Table 6). This result confirms that birds represent the main *Borrelia* reservoir at the study site. Our results, with respect to the prevalences of *A. phagocytophilum* and *B. lusitaniae*, also suggest that birds may not be available as hosts to questing tick cohorts during certain parts of the season when ruminants and reptiles seem to serve as the main tick hosts. These findings are consistent with the results by Tarageľová et al. [16] who found for a montane habitat that *B. lusitaniae* could be locally dominant *Borrelia* genospecies under a shortage of tick hosts. 

*R. helvetica* and *R. monacensis* were recorded in 5.9% (28/477) of questing *I. ricinus* ticks with *R. helvetica* being the dominant *Rickettsia* species. This prevalence value is again at the lower range limit of *Rickettsia* prevalences detected in Slovakia and other European countries (0.5–66%) [42,43,44]. The role of vertebrates in the life cycle of *R. helvetica* is still unclear. Rickettsiae were identified in ticks feeding on rodents and birds [95,96,97,98], but also in blood and tissue samples from birds, rodents, roe deer, sika deer and wild boar [46,95,98,99,100]. *Rickettsia* spp. is transmitted transovarially and transstadially. We can only speculate that rickettsial infections of attached ticks are the result of either a vertical route of infection (transovarial and/or transstadial transmission) in ticks or a very short rickettsiaemia in hosts or ticks co-feeding. While *I. ricinus* can be considered as the most important reservoir host for Rickettsiae [100], we found that the occurrence of *R. helvetica* coincided with the occurrence of *B. afzelii* (Figure 4). Therefore, the enzootic cycle of *R. helvetica* may be related to that of rodent-associated *B. afzelii*. Moreover, our findings on a relatively low prevalence of *Rickettsia* and *B. burgdorferi* s.l. and a high prevalence of *A. phagocytophilum* are consistent with the results by Václav et al. [101]. Václav et al. [101] found that *Anaplasma* prevalence was negatively assotiated with the prevalences of *Rickettsia* and *Borrelia*. Thus, our study indicates that (1) *Rickettsia* and *Borrelia* circulate in similar hosts and (2) hosts responsible for *Anaplasma* circulation contribute not only to the dillution of *Borrelia* but also of *Rickettsia* pathogens in feeding ticks.

## 5. Conclusions

This study implies that structure and seasonality in tick host communities may have an important role in the transmission potential and seasonal dynamics of different *Borrelia* genospecies. While birds acted as the main alternative hosts for questing ticks, based on *B. garinii* and *B. valaisiana* prevalences, they were not able to compensate for the low abundance of small rodents generally serving as the major *Borrelia* reservoirs. The biotic effects imposed by the dominant tick host, roe deer, appear to have had the most influential role in tick propagation as well as the transmission or suppression of specific tick-borne pathogens at the study area. Distinct seasonality in the density of tick hosts, such as temperate-zone birds, has the potential to shape the phenology of different *Borrelia* genospecies within sites, particularly, as our study suggests, under low abundances of alternative small vertebrate hosts. We propose that more attention should be given to the role of temporally fluctuating host communities within sites in order to understand the circulation of tick-borne pathogens and the seasonality of infection risk.

## Figures and Tables

**Table 1:** Number of *I. ricinus* ticks collected from single bird species and *B. burgdorferi* s.l.- and *R. helvetica*-infection rates for the urban forest in Bratislava, Slovakia, 2011–2012.

Bird Species	Number of Birds	*B. burgdorferi* s.l.-Positive/Examined (%)	*R. helvetica*-Positive/Examined (%)
*Parus major*	18	28/255 (11%)	13/255 (5.1%)
*Sitta europaea*	5	3/9 (33.3%)	0/9 (0%)
*Turdus merula*	2	5/9 (55.6%)	0/9 (0%)
*Erithacus rubecula*	2	1/15 (6.7%)	0/15 (0%)
*Dendrocopos major*	2	0	0
*Parus montanus*	2	0/5 (0%)	0/5 (0%)
*Fringilla coelebs*	1	0	0
*Parus caeruleus*	1	0	0
*Muscicapa striata*	1	0/2 (0%)	0/2 (0%)
Total	34	37/295 (12.5%)	13/295 (4.4%)

**Table 2:** Prevalence of *B. burgdorferi* s.l. and *R. helvetica* in bird-feeding *I. ricinus* ticks for the urban forest in Bratislava, Slovakia, 2011–2012.

	*B. burgdorferi* s.l. Positive/Examined (%)	*R. helvetica* Positive/Examined (%)
larvae	34/275 (12.4%)	12/275 (4.4%)
nymphs	3/20 (15%)	1/20 (5%)
Total	37/295 (12.5%)	13/295 (4.4%)

**Table 3:** Prevalence of pathogens in questing *I. ricinus* ticks for the urban forest in Bratislava, Slovakia, 2011–2012.

	Total Examined Ticks	*B. burgdorferi* s.l. Positive (%)								*Rickettsia* sp. Positive (%)		*A.P.* Positive (%)
		Total	*B.G.*	*B.V.*	*B.A.*	*B.L.*	*B.B.*S.S.	*B.S.*	Mixed	*R.H.*	*R.M.*	
Nymphs	485	35 (7.21%)	12 (2.47%)	8 (1.65%)	6 (1.24%)	4 (0.83%)	2 (0.41%)	1 (0.21%)	2 (0.41%)	23 (4.75%)	0	23 (6%)
Adults	58	2 (3.38%)	1 (1.69%)	1 (1.69%)	0	0	0	0	0	4 (6.78%)	1 (1.69%)	3 (5.08%)
Total	543	37 (6.81%)	13 (2.39%)	9 (1.66%)	6 (1.1%)	4 (0.74%)	2 (0.37%)	1 (0.18%)	2 (0.37%)	27 (4.97%)	1 (0.18%)	32 (5.89%)

*B.G.*—*B. garinii*, *B.V.*—*B. valaisiana*, *B.A.*—*B. afzelii*, *B.L.*—*B. lusitaniae*, *B.B.*S.S.—*B. burgdorferi* s.s., *B.S.*—*B. spielmanii*, *R.H.*—*R. helvetica*, *R.M.*—*R. monacensis*, *A.P.*—*A. phagocytophilum*.

**Table 4:** Prevalence of *A. phagocytophilum* in questing *I.ricinus* ticks in Slovakia. Overall test for differences among all sites: G = 116.85, df = 9, *p* < 0.001.

Study Site (Type)	Year of Tick Collection	*A. phagocytophilum* in Questing *I. ricinus* Ticks	Author
Bratislava (urban forest)	2011–2012	5.9% (32/543)	This study
Fugelka (natural forest)	2011–2013	3.1% (71/2257) n.s.	[59]
Bratislava (urban forest)	2011–2013	7.2% (153/2117) n.s.	[59]
Košice (urban forest)	2010	4.5% (10/224) n.s.	[30]
Bardejov (suburban forest)	2008	1.7% (3/179) n.s.	[30]
Bratislava (suburban/urban)	2009–2012	4% (10/248) n.s.	[30]
Čermeľ (natural forest)	2011–2012	0.9% (2/220) *	[37]
Hýľov (natural forest)	2011–2012	0.8% (2/266) **	[37]
Košice (suburban forest)	2011–2012	1.1% (2/176) n.s.	[37]
Rozhanovce (natural forest)	2011–2012	0.6% (4/714) **	[37]

Asterisks inform about post-hoc pair-wise comparisons between the study site and other sites; **/* denote statistically significant differences at α = 0.01/0.05 after the Holm *p*-adjustment. n.s.: non-significant.

**Table 5:** Prevalence of *B. burgdorferi* s.l. in questing *I. ricinus* nymphs in Slovakia. Overall test for differences among all sites: G = 62.62, df = 7, *p* < 0.001.

Study Site (Type)	Year of Ticks Collection	*B.b*.s.l. Prevalence (%) in Questing *I. ricinus* Nymphs	Author
Bratislava (urban forest)	2011–2012	7.2% (35/485)	This study
Šúr (natural forest)	2001–2002	27.8% (57/205) **	[26]
Malacky (urban park)	2001–2002	14.4% (31/215) n.s.	[26]
Brzotín (natural forest)	2001–2002	20% (14/70) *	[26]
Biskupice (natural forest)	1999	21.5% (28/130) **	[87]
Malacky (urban park)	1999	18.7% (37/198) **	[87]
Záhorská Ves (natural forest)	1999	27.8% (15/54) **	[87]
Záhorská Ves (natural forest)	2000	19% (33/174) **	[87]

Asterisks inform about post-hoc pair-wise comparisons between the study site and other sites; **/* denote statistically significant differences at α = 0.01/0.05 after the Holm *p*-adjustment. n.s.: non-significant.

**Table 6:** Prevalence of *B. garinii* in bird-feeding *I. ricinus* ticks in Slovakia. Overall test: G = 47.00, df = 1, *p* < 0.001.

Study Site (Type)	Year of Tick Collection	*B. garinii* Prevalence in Bird-Feeding *I. ricinus* Ticks	Author
Bratislava (urban forest)	2011–2012	97% (36/37)	This study
All localities (natural forest)	2001–2002	44% (155/352) **	[26]

** Denotes statistically significant difference at α = 0.01.
